# Reproductive outcome and survival of common bottlenose dolphins sampled in Barataria Bay, Louisiana, USA, following the *Deepwater Horizon* oil spill

**DOI:** 10.1098/rspb.2015.1944

**Published:** 2015-11-07

**Authors:** Suzanne M. Lane, Cynthia R. Smith, Jason Mitchell, Brian C. Balmer, Kevin P. Barry, Trent McDonald, Chiharu S. Mori, Patricia E. Rosel, Teresa K. Rowles, Todd R. Speakman, Forrest I. Townsend, Mandy C. Tumlin, Randall S. Wells, Eric S. Zolman, Lori H. Schwacke

**Affiliations:** 1 National Oceanic and Atmospheric Administration, National Centers for Coastal Ocean Science, Hollings Marine Laboratory, Charleston, SC, USA; 2 National Marine Mammal Foundation, San Diego, CA, USA; 3 Western Ecosystems Technology, Inc., Cheyenne, WY, USA; 4 Chicago Zoological Society, c/o Mote Marine Laboratory, Sarasota, FL, USA; 5 National Oceanic and Atmospheric Administration, National Marine Fisheries Service, Pascagoula, MS, USA; 6 Industrial Economics, Inc., Cambridge, MA, USA; 7 National Oceanic and Atmospheric Administration, National Marine Fisheries Service, Lafayette, LA, USA; 8 National Oceanic and Atmospheric Administration, National Marine Fisheries Service, Office of Protected Resources, Silver Spring, MD, USA; 9 Bayside Hospital for Animals, Fort Walton Beach, FL, USA; 10 Louisiana Department of Wildlife and Fisheries, Baton Rouge, LA, USA

**Keywords:** bottlenose dolphin, *Tursiops truncatus*, *Deepwater Horizon*, oil spill, reproductive outcome, survival

## Abstract

Common bottlenose dolphins (*Tursiops truncatus*) inhabit bays, sounds and estuaries across the Gulf of Mexico. Following the *Deepwater Horizon* oil spill, studies were initiated to assess potential effects on these ecologically important apex predators. A previous study reported disease conditions, including lung disease and impaired stress response, for 32 dolphins that were temporarily captured and given health assessments in Barataria Bay, Louisiana, USA. Ten of the sampled dolphins were determined to be pregnant, with expected due dates the following spring or summer. Here, we report findings after 47 months of follow-up monitoring of those sampled dolphins. Only 20% (95% CI: 2.50–55.6%) of the pregnant dolphins produced viable calves, as compared with a previously reported pregnancy success rate of 83% in a reference population. Fifty-seven per cent of pregnant females that did not successfully produce a calf had been previously diagnosed with moderate–severe lung disease. In addition, the estimated annual survival rate of the sampled cohort was low (86.8%, 95% CI: 80.0–92.7%) as compared with survival rates of 95.1% and 96.2% from two other previously studied bottlenose dolphin populations. Our findings confirm low reproductive success and high mortality in dolphins from a heavily oiled estuary when compared with other populations. Follow-up studies are needed to better understand the potential recovery of dolphins in Barataria Bay and, by extension, other Gulf coastal regions impacted by the spill.

## 1. Introduction

The *Deepwater Horizon* (DWH) oil spill released approximately 4.9 million barrels of oil into the Gulf of Mexico, making it the worst marine oil spill in US history [1]. In the wake of the unprecedented oil release, multiple studies were initiated to assess potential toxicological effects on marine wildlife. Experimental studies are demonstrating the likely toxic effects from exposure to DWH oil; for example, developmental abnormalities in the lowest trophic level estuarine fish [2] and cardiotoxicity in large pelagic predators [3]. Observational studies have shown impacts on deep-sea coral and benthic communities [4,5].

The potential impact of the oil spill on Gulf of Mexico cetacean populations is also of concern, particularly in light of previously observed long-term population impacts on killer whales (*Orcinus orca*) following the *Exxon Valdez* oil spill [6]. In the year following the *Exxon Valdez* spill, 33% of one resident pod and 41% of a transient group were lost, with neither group recovering to pre-spill numbers even two decades later [6]. For the DWH spill, several studies investigated the potential impact on cetaceans, and particularly common bottlenose dolphins (*Tursiops truncatus*; hereafter referred to as dolphins) in bays and sounds of the northern Gulf of Mexico. Barataria Bay, Louisiana, was one of the most heavily oiled regions of the coast [7], and one study reported on the compromised health of dolphins from this area [8]. The dolphins were temporarily captured, underwent health assessments and were released on site in August 2011, approximately 1 year after the flow of oil had ceased. The sampled dolphins exhibited a high prevalence of moderate–severe lung disease, consistent with studies of humans and other animals exposed to petroleum-associated chemicals via ingestion, inhalation or aspiration (see [8] for discussion). Serum biochemical abnormalities and low measures of adrenal hormones (both cortisol and aldosterone) indicative of hypoadrenocorticism were also observed [8]. Based on the observed abnormalities, experienced marine mammal veterinarians gave approximately half of the sampled dolphins a guarded or worse prognosis for survival, while 17% received a poor or grave prognosis [8].

While the health assessment study documented severe and prevalent disease conditions in Barataria Bay dolphins post-spill, the question of how these disease conditions would affect reproductive success and survival remained. Reproductive success and survival must be quantified in order to assess the potential impacts at the population level. Therefore, intensive vessel-based monitoring was conducted to determine reproductive success and survival of the dolphins sampled during the 2011 health assessments. Here, we report the results from these monitoring surveys, in total spanning 47 months.

## 2. Material and methods

### (a) Health assessment and tagging

**Figure 1.**
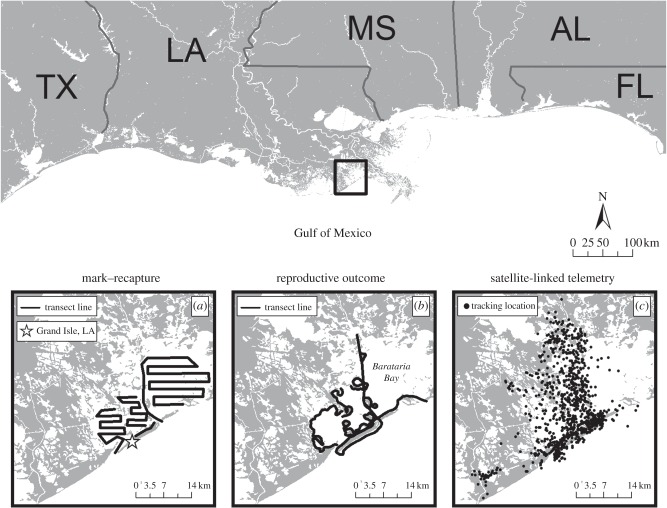
Map showing study area. Insets show (*a*) survey routes for photo-ID surveys for mark–recapture analysis, (*b*) survey routes for reproductive outcome surveys designed to provide coverage of areas encompassing previous sightings and satellite-linked locations of pregnant females, and (*c*) locations transmitted by satellite-linked tags (*n* = 25).

The Barataria Bay study area focused on estuarine waters near Grand Isle, LA, USA (29°14′ N, 90°00′ W; figure 1). The August 2011 capture–release health assessments of 32 dolphins have been previously described [8]. In order to facilitate recognition of individuals post-release, all dolphins received one to three digit freeze-brands on both sides of their dorsal fin following previously described methods [9]. Most dolphins (*n* = 30) were fitted with a satellite-linked tag (*n* = 4; SPOT-100 Single-point Finmount, 281A, Wildlife Computers, Redmond, WA, USA), a very high frequency (VHF) tag (*n* = 5; MM 130B VHF radio tag, Advanced Telemetry Systems, Inc., Insanti, MN, USA) or both (*n* = 21; table 1). Satellite-linked tags documented ranging patterns over several months following health assessment, while VHF tags facilitated re-acquisition of individuals for follow-up photographic monitoring. The electronic supplementary material details methods.

**Table 1.** Sighting histories and tag type for dolphins (*n* = 32) evaluated during August 2011 health evaluations in Barataria Bay, LA. The presence (1 or 2) or absence (0) of each animal was recorded for every month a survey was conducted. Outlined cells represent survey months in which satellite-linked data were received for animals with functioning tags. Bold numbers represent the final sighting of each individual. Odd/even field IDs represent females/males, respectively. Bold field IDs represent females that were pregnant at time of capture, as determined by ultrasonography. A number 2 indicates that the female was sighted with a calf. Month of estimated due date is shaded in grey. Sightings of mother/calf pairs following the due date were considered reproductive successes. Detection probability was estimated from the top CJS model *φ*_._, *p_t_*. 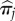 indicates the probability that the dolphin would be undetected for the number of contiguous occasions shown. n.a., not applicable. Tag types: S, satellite-linked; V, very high frequency (VHF); B, both VHF and satellite-linked.

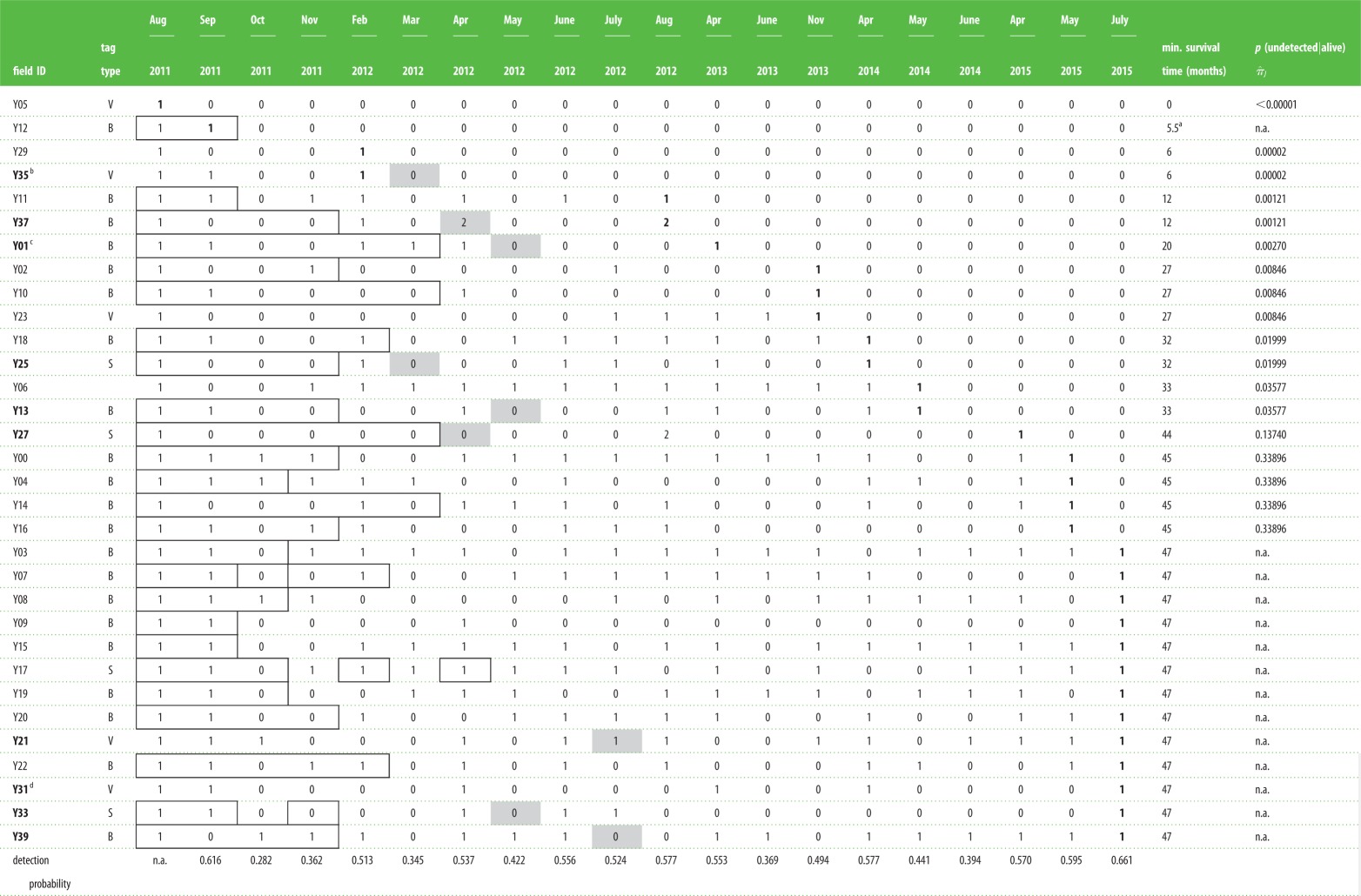

^a^Actual survival time based on carcass retrieval (31 Jan 2012).

^b^Y35 disappeared prior to her due date and presumably died along with her calf.

^c^Y01 was observed pushing a dead neonate in March 2013, 10 months after the expected due date (May 2012) of her 2011 pregnancy resulting in her second reproductive loss in 17 months.

^d^Ultrasonography of Y31 revealed the presence of a non-viable fetus; therefore, no due date is provided.

### (b) Vessel-based surveys

Data for analysis of reproductive outcome and survival were synthesized from differing types of vessel-based surveys conducted from September 2011 to July 2015 (see electronic supplementary material, figure S1). Surveys were frequent in the first year and included radio-tracking of tagged dolphins, photographic-identification surveys (photo-ID) specifically targeting the dolphins that had been ‘marked’ with freeze-brands during the health assessments, and reproductive outcome surveys specifically designed for follow-up of pregnant dolphins. In addition, photo-ID surveys targeting all dolphins in the Barataria Bay population for mark–recapture analysis had been ongoing prior to the health evaluations, and these surveys continued periodically through April 2014. Additional health evaluations were conducted in 2013 and 2014, targeting both new and previously captured individuals. We do not report here on the newly captured individuals from 2013 and 2014 due to lack of sufficient follow-up, but information from these surveys on presence/absence of dolphins from the 2011 sample cohort were included. The electronic supplementary material details varying survey types, with survey methods described in detail elsewhere [10].

### (c) Photo analysis

The digital photos of dolphin dorsal fins collected during surveys were analysed following standard photo-ID methods [10], wherein scars and dorsal-fin notches distinguish individual dolphins [11]. Captured dolphins were identified and matched using a combination of three methods: freeze-brands, tag placement and/or fin notches. Calves without distinguishing fin characteristics were matched via association with marked females (i.e. a dolphin whose length was no greater than 75% of the presumed mother's length and swimming in echelon position [12]). Two researchers verified all matches.

### (d) Satellite-linked location data

Satellite-linked locations were detected and processed by the Argos data collection and location system. Additional details can be found in the electronic supplementary material. Location maps were plotted using ArcMap v. 9.2 (ESRI, Redlands, CA, USA).

### (e) Data analysis

Individual resighting (‘capture’) histories over 47 months were compiled for the 32 dolphins sampled during the August 2011 study. Within a given month, multiple sightings of the same animal, regardless of survey type, were pooled to create one record. Capture histories consisted of 20 possible capture occasions, from August 2011 through July 2015 (table 1).

Two types of statistical analyses were conducted: (i) estimation of successful reproductive outcome for dolphins confirmed to be pregnant via ultrasound, and (ii) mark–recapture analysis to estimate an overall survival rate for the sample cohort.

#### (i) Estimated rate of reproductive success

Reproductive status at the time of health evaluation was determined by ultrasound examination, and biparietal skull diameter of the fetus was measured to estimate due date as previously described [13].

Calf survival was evaluated via photo-ID of the expectant female. Photographs of the expectant dolphin during sightings following her estimated due date were examined to assess presence of an associated calf. If a calf was sighted swimming alongside its presumed mother, the outcome was classified as a success. If an associated neonate/calf was absent two weeks following its estimated due date (to accommodate late deliveries) to 1 year after the latest estimated due date, the outcome was classified as a loss, either due to fetal or neonatal death. The proportion of successful outcomes was computed and compared with a published fetal survival success rate [13] using a Fisher's exact test.

#### (ii) Estimation of survival rate

A random-effects open-population Cormack–Jolly–Seber (CJS) model estimated survival rates of sampled dolphins [14,15]. CJS models condition on the initial capture of an individual, and model the survival (*φ*) and recapture probability (*p*) from that point forward. We hypothesized that each of *φ* and *p* could be constant or vary randomly through time, or that *p* might depend on the type of vessel-based survey (survey types = photo-ID, health evaluations, other). We fitted the following six CJS models to observed capture histories: random *φ* and random *p* [model (*φ_t_ p_t_*)], random *φ* and constant *p* [model (*φ*_t_ *p*_._)], constant *φ* and random *p* [model (*φ_._ p_t_*)], constant *φ* and *p* [model (*φ*_._ *p*_._)], random *φ* and *p* dependent upon survey type [model (*φ_t_ p*_survey_)], and constant *φ* and *p* dependent upon survey type [model (*φ*.*p*_survey_)]. To facilitate the variation in time between sampling occasions, time intervals were computed as the fraction of a year, based on months, between two occasions, and *φ_t_* was raised to the interval length during likelihood computation. This scaled *φ_t_* to a yearly time frame and facilitated modelling annual survival as constant. When modelling either *φ_t_* or *p_t_* as random, we estimated time variation using the random-walk models 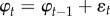 or 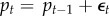.

Estimation was performed in R using the rjags package [16]. Code for all six models appears in the electronic supplementary material. We assumed uniform [0,1] priors when parameters were constant and for *φ*_1_ and *p*_1_. In random models, we assumed vague normal priors for 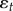 and 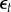 (i.e. 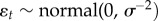, 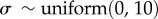). Priors for coefficients in the model relating survey type to capture probability were vague normals. The model's deviance information criterion (DIC) selected the best-fitting model [17].

The probability of non-detection for each dolphin over consecutive occasions assuming it was alive was also estimated in order to provide perspective for a given individual's disappearance. Using the recapture probabilities (*p_i_*) for each capture occasion *i* = 2–20 produced by the best-fitting model, the probability that a specific dolphin went undetected for *n* consecutive occasions after it was last seen, given that it was alive, was calculated as 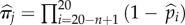.

## 3. Results

### (a) Satellite-linked tracking

The number of filtered locations for the 25 satellite-tagged dolphins ranged from 222 to 1067, while the total length of time that tags transmitted ranged from 48 to 260 days [18]. For those animals that were satellite-tagged, survey months in which satellite-linked data were received are indicated in table 1. Overall, the tagged dolphins did not exhibit long-range movements outside of Barataria Bay over the transmission period (figure 1).

### (b) Reproductive outcome of 2011 study animals

Ten females were confirmed pregnant through ultrasonography during the health evaluations. The gestation period for dolphins is approximately 12.5 months [19], and the pregnancies were all deemed as first or second trimester with estimated due dates ranging from March 2012 to July 2012 (table 1). One of the pregnancies (Y31) was determined to be non-viable at the time of ultrasound examination [8].

One of the pregnant dolphins, Y35, disappeared within six months of being sampled and presumably died. Y35 was last observed on 13 February 2012, approximately 16 days before her expected due date (table 1). She had been assigned a good prognosis when evaluated, although she had unusually low serum cortisol (0.92 µg dl^−1^) and aldosterone (below assay detection limit: 5.5 pg ml^−1^), indicative of hypoadrenocorticism; the adrenal hormone measures were not included in prognosis assignments due to this condition not having been previously reported in dolphins. Because she was not found on any of the 14 surveys following her due date, she is presumed dead, and her reproductive outcome was therefore classified as a loss.

Of the remaining confirmed pregnant dolphins, two (Y27 and Y37) were observed with calves following their due date, while six (Y01, Y13, Y21, Y25, Y33 and Y39) were observed without a calf following their due date (table 1). The reproductive success rate of 20% (95% CI: 2.50–55.6%) estimated for all confirmed pregnant dolphins was significantly lower than a previously reported pregnancy success rate of 83% for dolphins from Sarasota Bay, FL, USA [13] (Fisher's exact test, *p* < 0.01).

One dolphin (Y01), mentioned above, was observed pushing a neonate carcass on 10 March 2013, approximately 10 months after her May 2012 due date, a behaviour previously observed in females towards their dead calves [20]. Given an average 12.5-month gestation period, this indicates that Y01 conceived again around February or early March 2012. The observation of a neonate only 10 months after the expected due date of her 2011 pregnancy suggests that Y01 aborted her 2011 fetus prior to the end of her third trimester, allowing her to become pregnant again by May 2012, and that she had two consecutive reproductive failures.

### (c) Estimation of survival rate

The CJS mark–recapture survival model with constant *φ* and random *p* provided the best fit measured by DIC (table 2). The model yielded an apparent annual survival rate of 86.8% (95% CI: 80.0–92.7%) or annual mortality rate of 13.2%. Monthly detection probabilities, *p_t_*, ranged from 0.282 (October 2011) to 0.661 (July 2015) (table 1).

**Table 2.** DIC for each of the six fitted models. *φ*, survival; *p*, recapture probability.

model	DIC
*φ. p_t_*	729.1672
*φ_t_ p_**.**_*	734.4213
*φ_**.**_ p_t_*	747.3019
*φ_t_ p_t_*^a^	749.5216
*φ_t_ p*_survey_	760.7696
*φ_**.**_ p*_survey_	774.9495

^a^Inadequate mixing of MCMC chains.

Four of the 32 dolphins (12.5%), including Y35, disappeared within the first year of sampling and presumably died (table 1). All four went undetected for a minimum of 15 consecutive surveys and the estimated 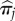 for each dolphin was less than 0.00001. Of the dolphins that disappeared, Y05 and Y12 each received a grave prognosis during their health evaluation, indicating the veterinary team expected imminent death. Both dolphins were diagnosed with severe lung disease, anaemia and poor body condition. Y05, a 16-year-old female, probably died within days of her evaluation. Although fitted with a VHF tag, researchers were unable to reacquire Y05 during the three months of radio-tracking or during any of the other subsequent monitoring surveys (table 1). Y12, a 16-year-old male, was found dead on Grand Isle Beach in January 2012. Necropsy results confirmed severe emaciation, and severe and chronic lung lesions, which probably contributed to his death. Y29, the remaining dolphin that disappeared within 1 year, had not been assigned a prognosis due to an incomplete health evaluation. She exhibited tachycardia (increased heart rate), began arching during her evaluation and was released early due to her apparent instability.

## 4. Discussion

The reproductive success rate for Barataria Bay dolphins was unexpectedly low (20%). In comparison, a similar study in Sarasota Bay that diagnosed dolphin pregnancies using the same methodology as in this study reported an 83% (95% CI = 0.52–0.99) pregnancy success rate [13]. Two of the cases were documented as fetal loss, one being the second trimester fetal death determined via ultrasound for Y31, and the other a presumed abortion of Y01's fetus prior to her third trimester. The timing of the loss for the remaining six pregnant animals could not be determined. Y35 disappeared and presumably died near her due date, but it is unknown whether she died just prior to parturition or whether she gave birth to a neonate that would have been unlikely to survive due to lack of maternal care. Five dolphins (Y13, Y21, Y25, Y33 and Y39) were sighted without calves approximately two to three months after their estimated due dates, a time period when mother–calf pairs are usually observed in close proximity on every surfacing [12]; therefore, we conclude these animals experienced either fetal or neonatal loss, but the exact timing cannot be determined.

The analysis of fetal skull measurements during ultrasound in August 2011 indicated conception dates ranging from March to July 2011, 7 to 13 months after the flow of DWH oil from the well had ceased. However, oil lingered in Barataria Bay long after the well was capped, and was still being cleaned from beaches and marshes throughout the duration of this study [7]. Potential routes of oil exposure include ingestion, inhalation, aspiration, dermal absorption or a combination thereof, during the height of the spill (summer 2010) and its aftermath, when oil and oil by-products were still present. Data from the satellite-linked tags demonstrated that sampled dolphins are resident to Barataria Bay with little to no movement beyond the bay; therefore, it is reasonable to conclude that the dolphins were exposed to DWH oil.

Numerous studies have reported reproductive effects following exposure to petroleum or petroleum-associated chemicals. Sea otters exposed to the *Exxon Valdez* oil spill experienced high rates of maternal, fetal and neonatal loss [21]. Mink experimentally exposed to crude and bunker C oil during pregnancy and lactation had few offspring and poor neonatal survival [22]. Exposure to crude oil or petroleum-related compounds in laboratory animals and humans has been associated with increases in spontaneous abortions [23] as well as increased mortality following birth [22]. Unfortunately, the literature lacks studies focusing on the long-term impact of petroleum products on reproduction [23].

Additionally, there are links among oil and hydrocarbon exposure, poor maternal health, and perinatal loss for dolphins. Schwacke *et al*. [8] noted the presence of a number of relatively uncommon disease conditions in the sampled animals consistent with effects observed in other mammals following oil and petroleum hydrocarbon exposure. Of the reproductive failures, over half had been diagnosed with moderate–severe lung disease (four of seven that had been given a lung disease score, or 57%), while neither of the dolphins that successfully calved had more than minor pulmonary concerns. In humans, uncontrolled pulmonary disease can cause complications during pregnancy and increase the risk of both maternal and fetal mortality [24]. In addition, low adrenal hormones in the face of capture stress were found in several of the sampled animals and interpreted as evidence of hypoadrenocorticism [8], a life-threatening condition if left untreated [25]. Y35 had evidence of hypoadrenocorticism and disappeared just prior to her expected due date, raising the question of whether disease complications could have led to her death either during or immediately following parturition. In humans with hypoadrenocorticism, pregnancy and parturition are of increased concern due to the risk of adrenal crisis, which has been correlated with increases in both fetal and maternal mortality [26]. Postpartum adrenal crises were reported in 55% of women diagnosed with adrenal insufficiency in an epidemiological study in the 1940s, prior to the introduction of synthetic steroids for treatment [27].

Reproductive pathogens, such as *Brucella*, can also cause reproductive impairment. *Brucella*, a known dolphin intracellular pathogen, can cause late-term abortions, stillbirths and weak calves [28]. Recent studies have documented the presence of *Brucella* in a number of US dolphin populations (D. Fauquier 2014, unpublished data) [29]. Immunotoxicity is a likely consequence of marine mammals exposed to the DWH oil spill [30] and could increase susceptibility to reproductive pathogens such as *Brucella*. Furthermore, the general poor maternal health documented in the sampled dolphins could impair their immune systems and contribute to an increased vulnerability to reproductive pathogens. Though the prevalence of brucellosis based on histopathology and/or polymerase chain reaction assays in non-perinatal dolphins found stranded in and around Barataria Bay did not differ significantly from reference groups [31], *Brucella* sp. can cause reproductive loss or abortion and not lead to severe clinical disease in the pregnant female [32].

Other chemical and biological toxins unrelated to the DWH spill have also been linked to reproductive impairment. Exposure to persistent organochlorine pollutants (POPs), specifically polychlorinated biphenyls and pesticides, has been linked to reproductive failure [33]. POPs are a particular concern for cetaceans as these lipophilic compounds accumulate in fatty tissues and magnify up the food chain to top marine predators. However, concentrations of a broad suite of POPs, including 45 polychlorinated biphenyls, 22 pesticides and 11 brominated compounds, were measured in Barataria Bay dolphins and found to be low in comparison with concentrations reported from dolphins in other areas of the US coast [8,34]. Some marine biotoxins have also been linked to reproductive failure. Specifically, abortions and premature parturition have been reported for California sea lions exposed to domoic acid, a toxin from marine diatoms in the genus *Pseudo-nitzschia* [35]. However, only a few stranded dolphins sampled during the first years of the northern Gulf of Mexico unusual mortality event (UME) through early 2012 have tested positive for domoic acid (8.6%) or brevetoxin (12%), another common marine biotoxin, and the levels measured have been consistent with background exposures [36]. Therefore, it is unlikely that POP or marine toxin exposure are underlying causes of the reproductive failures in Barataria Bay dolphins.

It is difficult to determine specific mechanisms of reproductive impairment in observational wildlife studies. A recent study in seabirds showed a long-term reduction in reproductive success during the 10 years following the *Prestige* oil spill [37]. However, whether the continued reproductive impairment in the seabirds is related to direct toxic effects of lingering oil exposure or other factors related to the spill, such as reduced food availability, was not determined. A similar situation exists for Barataria Bay dolphins; whether the observed reproductive failures are directly related to oil exposure or indirectly related to the oil through a cascade of other health impacts to the adult females, cannot currently be determined. However, given the documented poor health of Barataria Bay dolphins and the associated increased mortality demonstrated both by this study and by the increased strandings of dead dolphins that began after the DWH spill in and around Barataria Bay [38], it is unsurprising to find impacts on reproduction as well. It is unknown how long the increased reproductive failures presumably attributable to oil exposure will persist.

In addition to the high rate of reproductive failure, the Barataria Bay dolphins examined during the 2011 health evaluations demonstrated poor survival, with an apparent annual survival rate of only 86.8% (95% CI: 80.0–92.7%). In comparison, a mark–recapture study of dolphins near Charleston, SC, USA, reported an apparent annual survival rate of 95.1% (95% CI: 88.2–1.00%) [39], and a long-term photo-ID study in Sarasota Bay, FL, USA, reported a 96.2% survival rate [40]. Given the demonstrated site fidelity of the study population to Barataria Bay and unlikelihood that a dolphin would permanently leave the area, this study indicates an excess mortality of 8–9% annually for Barataria Bay dolphins above expected baseline based on other bay, sound and estuary stocks in the southeast USA.

Decreased survivorship is consistent with the disease conditions previously described for the sampled dolphins, including significant pulmonary disease, adrenal gland abnormalities and poor body condition, particularly given that these conditions would be likely to progress in the absence of medical treatment [25,41]. The high mortality rate is also consistent with the reported cluster of increased strandings of dead dolphins in and near Barataria Bay following the DWH oil spill [38]. The ongoing investigation of a UME of dolphins in the northern Gulf of Mexico identified a distinct cluster of increased strandings in and near Barataria Bay from August 2010 through December 2011 as compared with historical baseline, and strandings in the Barataria Bay area remained above average intermittently at least through the end of the reported period (June 2013) [38].

The dolphins that disappeared within the first year almost certainly represent mortalities. All of the dolphins that disappeared within the first year went undetected for 15 or more consecutive surveys and each had a probability of less than 0.00001 of being alive but undetected for this many survey occasions. Permanent emigration from the survey area is also unlikely given the lack of long-range movements determined via the satellite tracking (figure 1) and the lack of subsequent sightings of these freeze-branded dolphins at other northern Gulf of Mexico sites where photo-ID studies were occurring. Most of the dolphins sampled (78%) were known individuals from the Barataria Bay photo-ID catalogue prior to their sampling in August 2011. This further supports the hypothesis that these dolphins were long-term residents of Barataria Bay, unlikely to permanently emigrate, and if they had been in the area, should have been readily sightable.

Only one carcass (Y12) was recovered, but this is not surprising, as cetacean mortalities most often go unobserved [42]. Williams *et al*. [43] estimated that on average carcasses are recovered for only 2% of cetacean deaths in the northern Gulf of Mexico, although this average was for offshore cetacean species, not bottlenose dolphins. Even in a coastal area with high population density and heavy vessel traffic with a well-established local stranding response network, as is the case for Sarasota Bay, FL, USA, only about one-third of losses of long-term resident dolphins are recovered as carcasses [44]. Barataria Bay is much less developed, has less vessel activity than Sarasota Bay, and includes expanses of marsh and small islands where carcasses could easily remain undetected; therefore, it is expected that a high proportion of dolphin mortalities in Barataria Bay are unobserved.

## 5. Conclusion

In the wake of the DWH oil spill, dolphins living in one of the most heavily oiled bays along the Gulf of Mexico coast were documented with severe and highly prevalent disease conditions, raising significant concerns for potential long-term consequences for the population [8]. Our findings from follow-up monitoring studies confirm significant decreases in reproductive success and high mortality rates when compared with other populations not impacted by the spill. This evidence suggests that dolphin reproduction and survival is being impacted by chronic disease, indicating that the effects of the DWH oil spill have been long-lasting. Continued studies are needed to further understand the potential recovery trajectory for dolphins in Barataria Bay, as well as other Gulf Coast regions impacted by the spill.

## Supplementary Material

Technical Details for Survey and Data Analysis Methods
