# Supplementary material for: Reproductive outcome and survival of common bottlenose dolphins sampled in Barataria Bay, Louisiana, USA, following the Deepwater Horizon oil spill
Source: Proc Biol Sci. 2015 Nov 7;282(1818):20151944. doi: 10.1098/rspb.2015.1944 (PMC4650159; doi:10.1098/rspb.2015.1944)
Supplement: Technical Details for Survey and Data Analysis Methods [file rspb20151944supp1.pdf]

Technical Details for Survey and Data Analysis Methods

Reproductive Outcome and Survival of Common Bottlenose Dolphins Sampled in Barataria Bay, Louisiana, USA Following the *Deepwater Horizon* Oil Spill

Suzanne M. Lane[a], Cynthia R. Smith[b], Jason Mitchell[c] , Brian C. Balmer[a,d], Kevin P. Barry[e], Trent McDonald[c] , Chiharu S. Mori[f], Patricia E. Rosel[g], Teresa K. Rowles[h], Todd R. Speakman[a], Forrest I. Townsend[i], Mandy C. Tumlin[j], Randall S. Wells[c], Eric S. Zolman[a], Lori H. Schwacke

Methods

Satellite-linked and VHF Tagging

Seven dolphins were given a single tag in order to reduce handling time due to very poor health condition, unstable behavior during processing (*e.g.* abnormal heart rate and/or abnormal respirations), and/or later term pregnancy (2nd trimester or later). Two dolphins were outfitted with a single tag due to the small size of their dorsal fin. Two additional dolphins were not tagged at all: one because its dorsal fin was severely damaged from previous human interaction (entanglement and boat-strike), while the other was released prior to complete processing due to increasingly unstable behavior. Attachment of VHF and/or satellite-linked transmitters followed methods as described by Balmer *et al.* [1]. The satellite-linked tags were set to transmit during two four-hour 'windows' each day (02:00-05:59 and 08:00-11:59 local), based on ARGOS satellite pass prediction values, looking for satellites with >20° elevation for at least three minutes. Based on programmed transmission times and anticipated battery life, the tags were expected to transmit up to 240 tracking days.

The Argos DCS uses multiple, polar-orbiting satellites to receive data from Argos tags and transmits these data to ground-based processing centers [2]. Tag locations were calculated using the Doppler Effect on transmission frequency and a location processing algorithm (Kalman

filtering) to provide higher numbers of accurate locations. Data were categorized into location classes (LCs) based upon estimated error: LC3, <250m; LC2, 250m-500m; LC1, 500m-1500m; LC0, >1500m. Only the highest LCs (LC3 and LC2) were used for plotting tagged animal locations.

Vessel-based surveys

Photographic-identification surveys for mark-recapture analysis

In June 2010, intensive photo-ID surveys for mark-recapture analysis were initiated in Barataria Bay. Mark-recapture photo-ID surveys have been used extensively to estimate abundance and determine survivorship of small cetaceans within a given region [3-5]. The mark-recapture photo-ID surveys in Barataria Bay were based on a robust design [6], and each primary session included three traversals of a defined survey route [4] (Figure 1). In all, ten primary sessions were conducted from June 2010 through April 2014, six of which followed the 2011 health evaluations.

Radio-tracking and freeze brand monitoring surveys

Surveys were initiated in September 2011 specifically to re-sight dolphins sampled during the preceding health evaluations. The objective of these surveys was to maximize the chance of encountering one or more of the 32 sampled dolphins by focusing survey effort in areas utilized by the sampled animals. *Ad hoc* survey routes were determined daily based on weather, sighting conditions, recent satellite-linked transmitter locations, and sightings of freeze-branded individuals from prior photo-ID surveys.

Surveys, each covering three days, were conducted at three, five, and ten weeks post capture-release. Two, 4-element mast-mounted Yagi antennas [Advanced Telemetry Systems (ATS),

Isanti, MN, USA] attached to ATS R2000 scientific receivers were used to scan all tagged animal frequencies at 60-second intervals. Once a tag signal was received, observers on the tracking vessel would attempt to visually locate the tagged animal, obtain photos of it along with any associated dolphins, and record a GPS position. The goal within each three-day tracking interval was to visually locate and obtain photos of all tagged dolphins.

Additional monitoring surveys were conducted following the expected transmission life of the VHF tags, with the primary objective to monitor tag migration and loss [1]. Animals were identified by the freeze brands applied during the health evaluations. These survey sessions, each lasting 3-5 days, were conducted in February, March, April, and May 2012.

Reproductive outcome surveys

Surveys specifically designed to document reproductive outcomes, utilizing photo-ID survey methods as described above and in greater detail by Melancon *et al.* [7], were conducted over one-week periods in June, July, and August 2012. The objective of these surveys was first to locate pregnant females from the August 2011 health evaluations (determined via ultrasound), and second to document the presence or absence of an accompanying neonate as they were expected to give birth in spring 2012. Survey routes were designed to provide coverage of areas encompassing previous sightings and recent satellite-linked locations of the targeted females. Additional surveys were conducted in April 2014, May 2014, May 2015 and July 2015.

R code to estimate CJS models

Data Preparation:

The following code sets up data for later MCMC sampling, and is common to all models. Here, H32 is the 32 X 20 matrix of capture histories (Table 1). I is a vector of interval lengths between occasions, computed as fractions of a year (I = [1 1 1 3 1 1 1 1 1 1 8 2 5 5 1 1 10 1 2] / 12). survHealth and survMRecap are matrices of indicators for Health Evaluation and Photo-ID survey types. (survHealth[1,] = [0 0 0 0 0 0 0 0 0 0 0 0 1 0 0 0 1 0 0 0], survMRecap[1,] = [0 0 0 1 1 0 1 0 0 0 0 1 0 1 1 0 0 0 0 0]).

```
F.fix.censors <- function(H){
  # Replace occasions after '2' in capture history with NA's.
  for( i in 1:nrow(H)){
    if( sum(H[i,]==2, na.rm=T) > 0){
      col.with.2 <- (1:ncol(H))[H[i,]==2]
      H[i,(col.with.2+1):ncol(H)] <- NA
      H[i,col.with.2] <- 1
    }
  }
  H
}
ch <- F.fix.censors(H32)

#   Vector of first encounters
get.first <- function(x) min(which(x!=0))
f <- apply(ch,1,get.first)

#   Toss individuals first seen at last occasion (no information for CJS)
ch <- ch[f!=ncol(ch),]
f <- f[f!=ncol(ch)]

sHealth <- survHealth[1,]
sMRecap <- survMRecap[1,]
ints <- I
ns <- ncol(ch)
nan <- nrow(ch)
```

Utility function to execute MCMC chains and compute DIC:

The following function is a copy of the coda.samples function contained in rjags, with the addition of DIC computation and modification to return a list object.

```
coda.samples.dic <- function (model, variable.names = NULL, n.iter, thin = 1,
...)
```

```
{
  load.module('dic') # necessary for pD and deviance monitor

  start <- model$iter() + thin
  varnames=c(variable.names, c('deviance', 'pD'))
  out <- jags.samples(model, varnames, n.iter, thin,
                      type = "trace", ...)
  deviance <- out$deviance
  pD <- out$pD
  out$deviance <- NULL
  out$pD <- NULL
  nch<-model$nchain()
  ans <- vector("list", nch)
  for (ch in 1:nch) {
    ans.ch <- vector("list", length(out))
    vnames.ch <- NULL
    for (i in seq(along = out)) {
      varname <- names(out)[[i]]
      d <- dim(out[[i]])
      if (length(d) < 3) {
        stop("Invalid dimensions for sampled output")
      }
      vardim <- d[1:(length(d) - 2)]
      nvar <- prod(vardim)
      niter <- d[length(d) - 1]
      nchain <- d[length(d)]
      values <- as.vector(out[[i]])
      var.i <- matrix(NA, nrow = niter, ncol = nvar)
      for (j in 1:nvar) {
        var.i[, j] <- values[j + (0:(niter - 1)) * nvar +
                             (ch - 1) * niter * nvar]
      }
      vnames.ch <- c(vnames.ch, rjags:::coda.names(varname, vardim))
      ans.ch[[i]] <- var.i
    }
    ans.ch <- do.call("cbind", ans.ch)
    colnames(ans.ch) <- vnames.ch
    ans[[ch]] <- mcmc(ans.ch, start = start, thin = thin)
  }
  dic <- mean(as.vector(deviance)) + .5*var(as.vector(deviance))
  return(list(samples=mcmc.list(ans), dic=dic))
}
```

JAGS code and execution:

The following is JAGS code used to fit the {constant,constant} model.

```
require(rjags)

modelString = "
model {
  # Survival: Constant
  #  Capture: Constant
```

```
  # Priors:
  mean.phi ~ dunif( 0, 1 )
  mean.p ~ dunif(0,1)

  # Constraints:
  for( i in 1:nan ){
    for( j in f[i]:(ns-1)){
      phi[i,j] <- mean.phi^ints[j]
      p[i,j] <- mean.p
    }
  }

  # Likelihood:
  for( i in 1:nan ){
    # Latent state at first encounter
    z[i,f[i]] <- 1
    for ( j in (f[i]+1):ns ) {
      # State process
      phi.eff[i,j] <- z[i,j-1]*phi[i,j-1]
      z[i,j] ~ dbern(phi.eff[i,j])

      # Observation process
      p.eff[i,j] <- z[i,j] * p[i,j-1]
      ch[i,j] ~ dbern( p.eff[i,j] )
    }
  }
}
"

# Write the modelString to a file, using R commands:
.temp = file("model.txt","w") ;
writeLines(modelString,con=.temp) ;
close(.temp)
#----------------------------------------------------------------------------
dataList = list(
  ch = ch ,
  f = f,
  ints = ints,
  ns = ns,
  nan = nan
)
#-----------------------------------------------------------------------------
# Initial values
z = matrix(NA, nan, ns)
for(i in 1:nan) {
  indx = which(ch[i,]==1)
  if( max(indx) > f[i]){
    z[i,(f[i]+1):max(indx)] = 1
  }
}
initsList = list(z = z, mean.phi=0.85, mean.p=.5)
#-----------------------------------------------------------------------------
--
# Run chains.
parameters = c( "mean.phi", "mean.p" )
adaptSteps = 2000
burnInSteps = 15000
```

```
nChains = 3
numSavedSteps=2000
thinSteps=1
nIter = ceiling( ( numSavedSteps * thinSteps ) / nChains )

source("coda.samples.dic.r")  # routine to compute dic, returns mcmc.object
jagsModel = jags.model( "model.txt" , data=dataList ,  inits=initsList ,
                        n.chains=nChains , n.adapt=adaptSteps )
cat( "Burning in the MCMC chain...\n" )
update( jagsModel , n.iter=burnInSteps )
cat( "Sampling final MCMC chain...\n" )
samples = coda.samples.dic( jagsModel , variable.names=parameters ,
                            n.iter=nIter , thin=thinSteps )
```

Other models:

Following are JAGS model statements for the remaining 5 models. Remaining code for data, initial values, and model executions is nearly identical to the above with minor modification.

```
model {
  # Survival: Random Walk
  #  Capture: Constant

  # Priors:
  PHI[1] ~ dunif( 0, 1 )
  mean.p ~ dunif(0,1)
  sigma.phi ~ dunif(0,10)

  # Constraints:
  mu.phi[1] <- log(PHI[1]/ (1-PHI[1]))
  for(j in 2:(ns-1)){
    mu.phi[j] <- mu.phi[j-1] + epsilon[j]
    logit(PHI[j]) <- mu.phi[j]
  }
  tau <- pow(sigma.phi,-2)

  for( i in 1:nan ){
    for( j in f[i]:(ns-1)){
      phi[i,j] <- PHI[j]^ints[j]
      p[i,j] <- mean.p
    }
  }
  for(j in 2:(ns-1)){
    epsilon[j] ~ dnorm(0,tau)
  }


  # Likelihood:
  for( i in 1:nan ){
    # Latent state at first encounter
    z[i,f[i]] <- 1
    for ( j in (f[i]+1):ns ) {
      # State process
      phi.eff[i,j] <- z[i,j-1]*phi[i,j-1]
      z[i,j] ~ dbern(phi.eff[i,j])
```

```
      # Observation process
      p.eff[i,j] <- z[i,j] * p[i,j-1]
      ch[i,j] ~ dbern( p.eff[i,j] )
    }
  }
}
------------------------------------------------------------
model {
  # Survival: Constant
  #  Capture: Random Walk

  # Priors:
  P[1] ~ dunif( 0, 1 )
  mean.phi ~ dunif(0,1)
  sigma.p ~ dunif(0,10)

  # Constraints:
  mu.p[1] <- log(P[1]/ (1-P[1]))
  for(j in 2:(ns-1)){
    mu.p[j] <- mu.p[j-1] + epsilon[j]
    logit(P[j]) <- mu.p[j]
  }
  tau <- pow(sigma.p,-2)

  for( i in 1:nan ){
    for( j in f[i]:(ns-1)){
      phi[i,j] <- mean.phi^ints[j]
      p[i,j] <- P[j]
    }
  }
  for(j in 2:(ns-1)){
    epsilon[j] ~ dnorm(0,tau)
  }


  # Likelihood:
  for( i in 1:nan ){
    # Latent state at first encounter
    z[i,f[i]] <- 1
    for ( j in (f[i]+1):ns ) {
      # State process
      phi.eff[i,j] <- z[i,j-1]*phi[i,j-1]
      z[i,j] ~ dbern(phi.eff[i,j])

      # Observation process
      p.eff[i,j] <- z[i,j] * p[i,j-1]
      ch[i,j] ~ dbern( p.eff[i,j] )
    }
  }
}
------------------------------------------------------------
model {
  # Survival: Random Walk
  #  Capture: Random Walk

  # Priors:
```

```
  P[1] ~ dunif( 0, 1 )
  PHI[1] ~ dunif(0,1)
  sigma.phi ~ dunif(0,10)
  sigma.p ~ dunif(0,10)

  # Constraints:
  mu.phi[1] <- log(PHI[1]/ (1-PHI[1]))
  mu.p[1] <- log(P[1]/ (1-P[1]))

  for(j in 2:(ns-1)){
    mu.phi[j] <- mu.phi[j-1] + epsilon.phi[j]
    logit(PHI[j]) <- mu.phi[j]
    mu.p[j] <- mu.p[j-1] + epsilon.p[j]
    logit(P[j]) <- mu.p[j]
  }

  tau.phi <- pow(sigma.phi,-2)
  tau.p <- pow(sigma.p,-2)

  for(j in 2:(ns-1)){
    epsilon.phi[j] ~ dnorm(0,tau.phi)
    epsilon.p[j] ~ dnorm(0,tau.p)
  }

  for( i in 1:nan ){
    for( j in f[i]:(ns-1)){
      phi[i,j] <- PHI[j]^ints[j]
      p[i,j] <- P[j]  # Actually, capture prob at occasion j+1
    }
  }


  # Likelihood:
  for( i in 1:nan ){
    # Latent state at first encounter
    z[i,f[i]] <- 1
    for ( j in (f[i]+1):ns ) {
      # State process
      phi.eff[i,j] <- z[i,j-1]*phi[i,j-1]
      z[i,j] ~ dbern(phi.eff[i,j])

      # Observation process
      p.eff[i,j] <- z[i,j] * p[i,j-1]  # p[i,j-1] = P(capture at occasion j)
      ch[i,j] ~ dbern( p.eff[i,j] )
    }
  }
}
------------------------------------------------------------
model {

  # Survival: Random Effects
  #  Capture: Survey type

  # Priors:
  mu.mean.phi ~ dnorm( 2, 3.5 )
  sigma ~ dunif(0,10)
  for(i in 1:3){
```

```
    beta[i] ~ dnorm(0,10)
  }

  # Constraints:
  tau <- pow(sigma,-2)
  for(j in 1:(ns-1)){
    mu.phi[j] ~ dnorm( mu.mean.phi, tau )
    logit(PHI[j]) <- mu.phi[j]
  }

  for(j in 1:(ns-1)){
    mu.p[j] <- beta[1] + beta[2]*sHealth[j] + beta[3]*sMRecap[j]
  }

  for( i in 1:nan ){
    for( j in f[i]:(ns-1)){
      phi[i,j] <- PHI[j]^ints[j]
      logit(p[i,j]) <- mu.p[j]
    }
  }


  # Likelihood:
  for( i in 1:nan ){
    # Latent state at first encounter
    z[i,f[i]] <- 1
    for ( j in (f[i]+1):ns ) {
      # State process
      phi.eff[i,j] <- z[i,j-1]*phi[i,j-1]
      z[i,j] ~ dbern(phi.eff[i,j])

      # Observation process
      p.eff[i,j] <- z[i,j] * p[i,j-1]
      ch[i,j] ~ dbern( p.eff[i,j] )
    }
  }
}
------------------------------------------------------------
model {

  # Survival: Constant
  #  Capture: Survey type

  # Priors:
  mu.mean.phi ~ dnorm( 2, 3.5 )
  for(i in 1:3){
    beta[i] ~ dnorm(0,10)
  }

  # Constraints:
  for(j in 1:(ns-1)){
    logit(PHI[j]) <- mu.mean.phi
  }

  for(j in 1:(ns-1)){
    mu.p[j] <- beta[1] + beta[2]*sHealth[j] + beta[3]*sMRecap[j]
  }
```

```
  for( i in 1:nan ){
    for( j in f[i]:(ns-1)){
      phi[i,j] <- PHI[j]^ints[j]
      logit(p[i,j]) <- mu.p[j]
    }
  }


  # Likelihood:
  for( i in 1:nan ){
    # Latent state at first encounter
    z[i,f[i]] <- 1
    for ( j in (f[i]+1):ns ) {
      # State process
      phi.eff[i,j] <- z[i,j-1]*phi[i,j-1]
      z[i,j] ~ dbern(phi.eff[i,j])

      # Observation process
      p.eff[i,j] <- z[i,j] * p[i,j-1]
      ch[i,j] ~ dbern( p.eff[i,j] )
    }
  }
}
```

Figure S1. Timing and types of surveys used in the survival and reproductive outcome analysis. Numbers above each symbol represent the number of surveys conducted in each month.

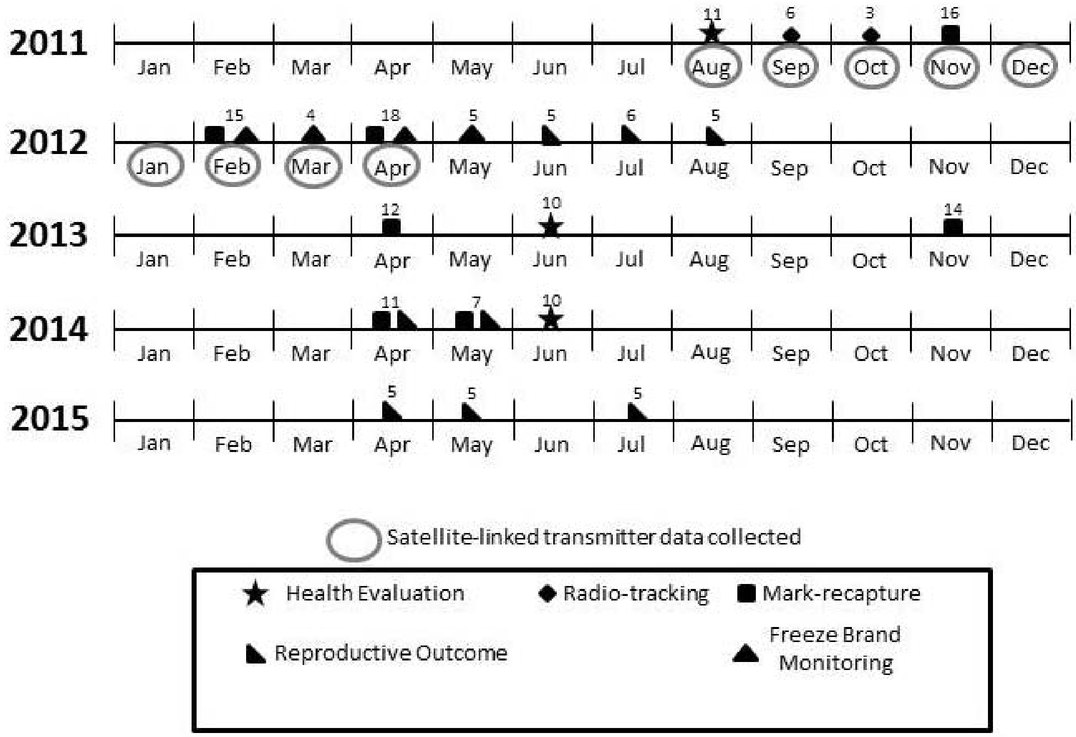

Table S1. Summary information for individual dolphins monitored following the 2011 health evaluations in Barataria Bay, LA. Criteria for overall prognosis, lung disease, and hematological and serum biochemical panels were defined in Schwacke *et al.* [8]. A "1" indicates the individual was classified as out-of-range for the given panel, "0" indicates the individual was within normal range. *Indicates the serum aldosterone was below assay detection limit (5.5 pg/mL).

									Hematological and Serum Biochemical Parameter Panels						
FB	Sex	Age (Years)	Pregnant	Length (cm)	Weight (kg)	Overall Prognosis	Poor Body Condition	Lung Disease	Inflammation Panel	Hypoglycemia	Iron Panel	Hepatobiliary Panel	Anemia	Aldosterone (pg/mL)	Cortisol (µg/dL)
Y05	F	16	0	251	154	grave	1	severe	1	1	0	1	1	5.5*	2.6
Y12	M	16		261	149	grave	1	severe	1	0	0	1	1	23.9	2.35
Y29	F		0	217					0	0	0	0	0	5.5*	2.69
Y35	F		1	224		good		normal	0	0	0	0	0	5.5*	0.92
Y11	F	19.5	0	253	173	guarded		severe	0	0	1	0	0	13.49	2.23
Y37	F		1	225		fair		mild	1	0	0	0	0	5.5*	0.2
Y01	F	42	1	243	143	good		normal	1	0	0	0	0	5.5*	1.59
Y02	M	17		263	201	guarded	0	mild	1	0	0	0	0	5.5*	0.34
Y10	M			258	191	poor	0	severe	0	1	1	0	0	5.5*	0.59
Y23	F	4	0	187	82	good	0	mild	0	0	0	0	0	5.5*	0.24
Y18	M			271	253	fair	0	mild	0	1	0	0	0	5.5*	0.93
Y25	F		1	239					0	0	0	0	0	13.22	0.27
Y06	M	4		210	114	fair	0	normal	0	0	0	0	0	5.5*	1.55
Y13	F	17	1	230	143	good		normal	0	0	1	0	0	117.46	2.74
Y27	F		1	239	183	good	0	mild	0	0	0	0	0	48.72	1.86
Y00	M	8		232	130	good	1	mild	0	0	0	0	0	27.13	0.82
Y04	M	21		255	182	fair	0	mild	0	0	0	0	0	67.07	2.43
Y14	M	5.75		216	120	fair	0	normal	1	0	0	0	0	189.11	3
Y16	M	21		273	169	guarded	1	mild	1	1	0	1	0	5.5*	0.83
Y03	F	6.5	0	221	119	guarded	0	moderate	1	0	0	0	0	91.16	2.19
Y07	F	5	0	199	105	guarded	0	normal	1	0	0	0	1	133.03	3.56
Y08	M	13		274	202		1		0	0	0	0	0	5.5*	0.4
Y09	F	22	0	241	148	fair	0	moderate	0	0	1	0	0	37.99	0.89
Y15	F	3.75	0	198	88	guarded	0	normal	0	0	0	1	0	181.18	5.38
Y17	F	5	0	209	92	guarded	0	mild	1	0	0	0	0	5.5*	1.96
Y19	F	6	0	227	117	good	0	normal	0	0	0	0	0	132.09	2.84
Y20	M	21		267	240	fair	0	mild	0	0	0	0	0	5.5*	0.45
Y21	F	16	1	234	158	poor		severe	0	1	0	0	0	25.69	1.22
Y22	M	12		234	150	fair	0	normal	1	1	1	1	0	38.93	1.37
Y31	F		1	239	175	poor	0	moderate	1	1	1	1	0	5.5*	1.78
Y33	F		1	228	156	guarded	0	moderate	0	0	0	0	1	5.5*	0.62
Y39	F		1	242		guarded		moderate	1	0	1	0	0	5.5*	0.34

References

1. Balmer B.C., Wells R.S., Howle L.E., Barleycorn A.A., McLellan W.A., Pabst D.A., Rowles T.K., Schwacke L.H., Townsend F.I., Westgate A.J., et al. 2014 Advances in cetacean telemetry: A review of single- pin transmitter attachment techniques on small cetaceans and development of a new satellite- linked transmitter design. *Mar Mamm Sci* **30**(2), 656-673.
2. Collecte Localisation Satellites. 2011 *Argos user's manual*. Largo, MD, USA, CLS America Inc.
3. Speakman T.R., Lane S.M., Schwacke L.H., Fair P.A., Zolman E.S. 2010 Mark-recapture estimates of seasonal abundance and survivorship for bottlenose dolphins (*Tursiops truncatus*) near Charleston, South Carolina, USA. *Journal of Cetacean Research and Management* **11**(2), 153-162.
4. Balmer B.C., Schwacke L.H., Wells R.S., Adams J.D., George R.C., Lane S.M., McLellan W.A., Rosel P.E., Sparks K., Speakman T., et al. 2013 Comparison of abundance and habitat usage for common bottlenose dolphins between sites exposed to differential anthropogenic stressors within the estuaries of southern Georgia, U.S.A. *Marine Mammal Science* **29**(2), E114-E135. (doi:10.1111/j.1748-7692.2012.00598.x).
5. Balmer B.C., Wells R.S., Nowacek S.M., Nowacek D.P., Schwacke L.H., McLellan W.A., Scharf F.S., Rowles T.K., Hansen L.J., Spradlin T.R., et al. 2008 Seasonal abundance and distribution patterns of common bottlenose dolphins (*Tursiops truncatus*) near St. Joseph Bay, Florida, USA. *Journal of Cetacean Research and Management* **10**(2), 157-167.
6. Kendall W.L., Nichols J.D., Hines J.E. 1997 Estimating temporary emigration using capture-recapture data with Pollock's robust design. *Ecology* **78**(2), 563-578.
7. Melancon R., Lane S.M., Speakman T., Hart L.B., Sinclair C., Adams J., Rosel P.E., Schwacke L. 2011 Photo-identification field and laboratory protocols utilizing FinBase version 2. (p. 46
8. Schwacke L.H., Smith C.R., Townsend F.I., Wells R.S., Hart L.B., Balmer B.C., Collier T.K., De Guise S., Fry M.M., Guillette L.J., Jr., et al. 2014 Health of common bottlenose dolphins (*Tursiops truncatus*) in Barataria Bay, Louisiana, following the *Deepwater Horizon* oil spill. *Environmental Science and Technology* **48**, 93-103. (doi:10.1021/es403610f).
